# Longitudinal analysis of subfoveal choroidal thickness after panretinal laser photocoagulation in diabetic retinopathy using swept-source optical coherence tomography

**Published:** 2020

**Authors:** Kamel Taher Eleiwa, Ahmed Bayoumy, Mahmoud Abdelrahman Elhusseiny, Khalid Gamil, Amr Sharawy

**Affiliations:** * Department of Ophthalmology, Faculty of Medicine, Benha University, Egypt; ** Bascom Palmer Eye Institute, University of Miami Miller School of Medicine, Miami, FL; *** Department of Ophthalmology, Faculty of Medicine, Cairo University, Egypt; **** Department of Ophthalmology, Boston Children’s Hospital, Harvard Medical School, USA

**Keywords:** choroidal thickness, diabetic retinopathy, panretinal photocoagulation

## Abstract

**Purpose:** To describe the central three-dimensional (3D) thickness profile of the macula (CMT) and the subfoveal choroidal region (SFCT) in diabetic retinopathy (DR) following panretinal laser photocoagulation (PRP) using swept-source optical coherence tomography (SS-OCT).

**Methods:** A prospective observational study including 17 eyes with proliferative DR (PDR) and 27 eyes with severe nonproliferative DR (sNPDR)] for whom PRP was done. All subjects received SS-OCT imaging before and 3 months after PRP (POM#3). SFCT and CMT changes were analysed at both visits. Intraclass Correlation Coefficients (ICC) and Coefficients of Variation (COV) were used to test the accuracy of thickness data.

**Results:** SFCT has thinned from 233 ± 54 µm before PRP treatment to 216 ± 51 µm 3 months later (p < 0.001). Likewise, CMT declined at POM#3 as compared to pre-PRP status (p<0.001). SFCT was thinner in PDR before and at POM#3 (p<0.05) than sNPDR; whereas, no significant difference was observed in CMT between both groups in the two visits. No significant changes were found between groups in SFCT and CMT at POM#3. Regarding reliability, ICCSFCT=0.98 and ICCCMT=0.99. The COVs for CMT and SFCT were 5.03% and 5.91%, respectively.

**Conclusion:** The mean SFCT and CMT decreased 3 months after PRP. We also reported reliability of SFCT measurements in DR using SS-OCT.

**Abbreviations:** SS = Swept-Source, TD = time domain, SD = spectral domain, FD = Fourier-domain, 3D = three-dimensional, 2D = two-dimensional

## Introduction

Diabetic retinopathy is a universal cause of vision loss [**1**]. Pan retinal laser photocoagulation (PRP) is an efficacious treatment for both severe non-proliferative (sNPDR) and proliferative (PDR) diabetic retinopathy [**2**]. Retinal and choroidal vascular changes have been reported. In literature, choroidal diabetic changes have been reported such as neovascularization [**5**], choriocapillaris degeneration [**3**], and prolonged choroidal vascular filling time [**4**]. In-vivo choroidal imaging has been made possible due to the developments in OCT technology. Although the PRP-induced changes in SFCT and CMT has been previously studied, the results were contradictory and many studies were conducted retrospectively [**6**-**13**]. The aim of our study was to prospectively analyze the changes in the CMT and SFCT 3 months post-PRP using SS-OCT. 

## Methods

**Study Population**

This study was approved by Benha University Institutional Review Board. All participants signed an informed consent before getting enrolled. The design of the study conformed with the Helsinki Declaration for Biomedical Research principles.

**Fig. 1:**
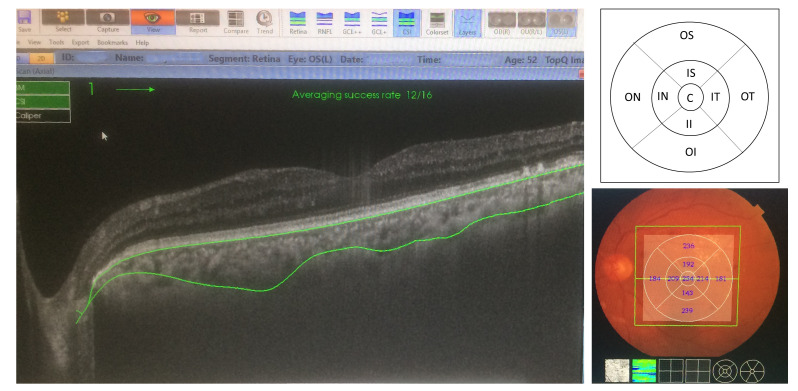
Swept source-OCT image of the retina (left image) of the left eye showing the automatic layer segmentation and the mean regional thickness values were automatically generated and divided into sections of the ETDRS map (bottom right). The SFCT was determined as the perpendicular distance between the outermost edge of the hyper-reflective line of the retinal pigment epithelium (RPE) and the choroidal-scleral interface (CSI) in the foveal region. Top right image shows the scheme of the ETDRS grid zones. C = centre; IS = inferior superior; OS = outer superior; II = Inner inferior; OI = outer inferior; IT = inner temporal; OT = outer temporal; IN = inner nasal; ON = outer nasal. PDR = Proliferative diabetic retinopathy; sNPDR = severe Non proliferative diabetic retinopathy; DME = Diabetic macular edema; SFCT = Subfoveal choroidal thickness; CMT = central macular thickness

Forty-four treatment-naive eyes (44 patients); 17 eyes with PDR, and 27 eyes with sNPDR, for whom PRP was indicated and performed [**14**], were prospectively enrolled from April 2017 to July 2018 in Benha Ophthalmology Department, Benha University Hospital, and Egyptian Eye Academy Centre. Upon the proposed categorization via the Global Diabetic Retinopathy Project Group, DR severity was graded [**15**]. 1200-1600 burns were applied using 532 nm doubled-YAG laser, per ETDRS guidelines [**16**,**17**]. Laser power, duration, and spot diameter were adjusted to 100 to 300 mW, 200-300 ms, and 200-300 microns respectively to make an optimal retinal burn. Having a CMT >300 µm before PRP [**18**,**19**], laser treatment was given per ETDRS protocol [**17**], at the judgment of a retina specialist. All candidates underwent thorough ocular examination at baseline and POM#3 including best-distant visual acuity (logMAR), pupillary light reflex, slit-lamp examination, intraocular pressure measurement (IOP) using Goldmann tonometry and dilated funduscopy. Using a 3D volumetric raster scan protocol, all patients underwent SS-OCT (DRI OCT Triton-plus; Topcon, Tokyo, Japan) after mydriasis [**20**], and in both visits [**21**]. This machine utilizes a 1050 nm wavelength light source with a 100,000 A Scans/ sec scanning speed with a 2.6 mm imaging depth [**22**]. For each patient, a three-dimensional (3D) volumetric data set was generated using 128 x 256 scanning sections covering a 6 mm x 6 mm area centered upon the fovea. The vertical distance from the retinal pigment epithelium to the choroido-scleral interface (CSI) at the fovea was determined as SFCT. Topcon Advanced Boundary Software was used to generate 3D thickness maps (**Fig. 1**) [**23**]. The mean retinal and choroidal thickness of the central 1 mm region corresponded to CMT and SFCT, respectively. Exclusion criteria were eyes with poor image qualities and eyes with ocular tension higher than 21 mmHg, or retinal disorders other than DR, or having history of ocular trauma, cataract surgery, intravitreal or sub-tenon injections, retinal laser surgery, and vitrectomy.

## Statistical Analysis

Statistical analyses were done using SPSS software version 26 to calculate descriptive statistics for all eyes. Paired t-test was used to compare CMT and SFCT at both visits. Comparisons between PDR and NPDR were done using Mann Whitney U-test for numerical data, and Chi-square test for categorical data. Pearson coefficients (R value) were used to test correlations between all thickness parameters and the clinical severity of DR. We concluded the precision of automatic measurements with the intraclass correlation coefficient (ICC) and coefficient of variation (COV). 

## Results

**Fig. 2:**
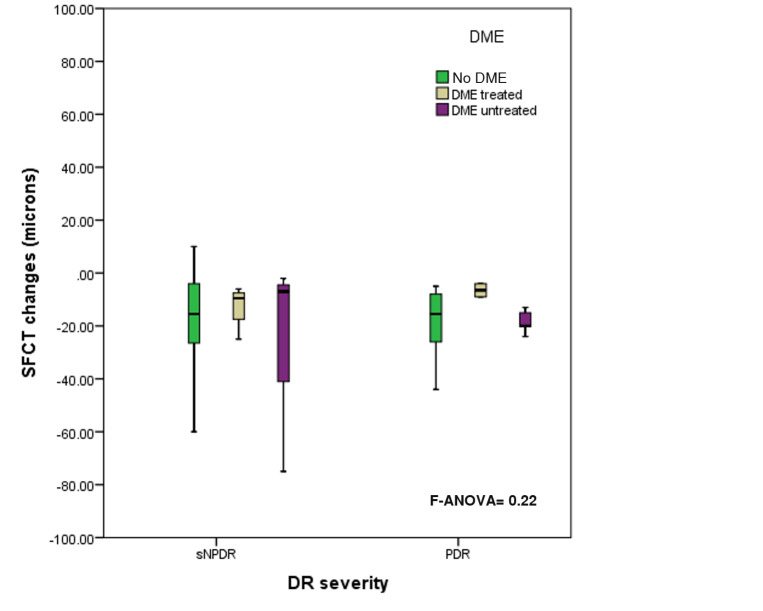
Box-plot distributions showing that there was no factorial effect of the existence of baseline DME on the variations in the average SFCT between both subgroups

The study cohort included 44 eyes of 44 subjects. The average postoperative follow-up period after the last session of the PRP was 3 ± 0.6 months (range, 2.6-4.5 months). The average patient age was 55.2 ± 12.1 years. All the patients were type 2 DM with a mean duration of 13 ± 3 years. Twenty-four eyes were of male patients (54.5%). The average preoperative BCVA (logMAR) was 0.4 (range: 0-2). sNPDR existed in 27 eyes (61.4%) (**Table 1**). Centre involving DME existed preoperatively in 14 eyes (31.8%). **Table 2** shows the demographic features of sNPDR and PDR subgroups. DME was found to have no significant effect on the changes in the SFCT between the two subgroups (P= 0.22, **Fig. 2**).

**Table 1:** Average preoperative BCVA

General characteristics (n= 44 eyes)		Values
Age (years)	Mean ± SD	55 ± 12
Gender	Males n (%)	24 (54.5)
	Females n (%)	20 (45.5)
HBA1c	Mean ± SD	10 ± 1
DM 2 duration (years)	Mean ± SD	13 ± 3
Diabetic retinopathy severity	Proliferative diabetic retinopathy n (%)	17 (38.6)
	Severe non proliferative diabetic retinopathy n (%)	27 (61.4)
Diabetic macular edema	n (%)	14 (31.8)
Visual acuity (LogMAR)	Median (range)	0.4 (0-2)
Baseline Subfoveal choroidal thickness (µm)	Mean ± SD	233 ± 54
Baseline central macular thickness (µm)	Mean ± SD	313 ± 95

**Table 2:** Demographic features of sNPDR and PDR subgroups

			Proliferative diabetic retinopathy (n = 17)	Severe non proliferative diabetic retinopathy (n = 27)	P- value
Age (years)		Mean ± SD	50 ± 14	59 ± 9	0.06
Gender		Males	9 (52.9)	15 (56)	0.41
		Females	8 (47.1)	12 (44)	
HBA1c		Mean ± SD	11 ±1	9 ±1	0.003
Visual acuity (LogMAR)		Median (range)	0.4 (0.1-2)	0.4 (0-1.6)	0.874
Central macular thickness	Pre	Mean ± SD	314 ± 87	312 ± 101	0.828
	At 3 months	Mean ± SD	285 ± 88	286 ± 82	0.758
	% Change	Median (range)	-7.1 (-23.1-2.4)	-7.1 (-23.1-2.4)	0.368
Subfoveal choroidal thickness	Pre	Mean ± SD	207 ± 48	249 ± 51	0.009
	At 3 months	Mean ± SD	192 ± 49	228 ± 49	0.036
	% Change	Median (range)	-16 (-44-4)	--19 (-75-10)	0.568
Mann Whitney U test was used. Chi-square test was used for gender.					

Automatic measurements demonstrated an excellent reliability for both CMT and SFCT with ICCSFCT=0.98 and ICCCMT=0.99. The COVs for CMT and SFCT were 5.03% and 5.91%, respectively. In the sNPDR subgroup, the average SFCT was thicker than the PDR group at both visits, while CMT showed no difference (**Table 2**). There was a significant drop of the mean CMT from a baseline of 313 ± 95 µm into 286 ± 83 µm 3 months post-PRP (p<0.001, **Fig. 3-A**). SFCT thinned from 233 ± 54 µm at 1st visit to 216 ± 51 µm at POM#3 (p<0.001, **Fig. 3-A**). 

In the subgroup of severe NPDR patients who underwent PRP (27 eyes), the mean CMT and SFCT were significantly reduced from the baseline of 312 ± 101 µm and 249 ± 51 µm to 286 ± 82 µm and 228 ± 49 µm 3 months after PRP, respectively (p <0.001, **Fig. 3-B**). The mean CMT and SFCT were reduced from 314 ± 87 µm and 207 ± 48 µm, to 285 ± 88 µm and 192 ± 49 µm 3 months in PRP-treated PDR eyes, respectively (p<0.001, **Fig. 3-B**).

**Fig. 3-A:**
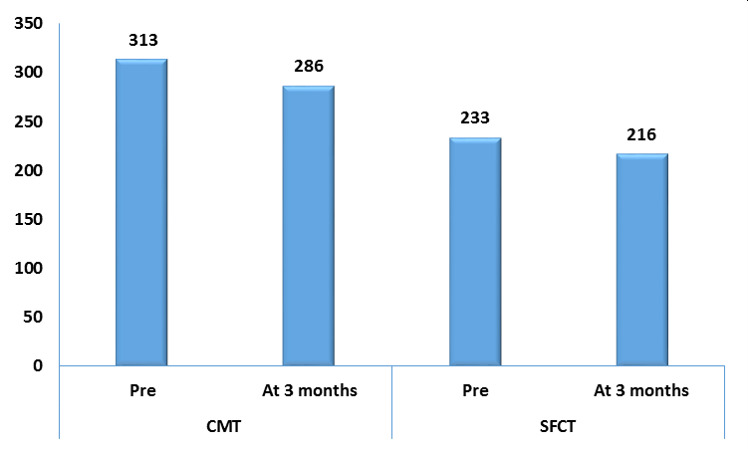
Bar chart showing that CMT decreased from 313 ± 95 µm before PRP to 286 ± 83 µm 3 months later (p<0.001). Also, the average SFCT was significantly reduced from 233 ± 54 µm at baseline to 216 ± 51 µm at 3 months (p<0.001)

**Fig. 3-B:**
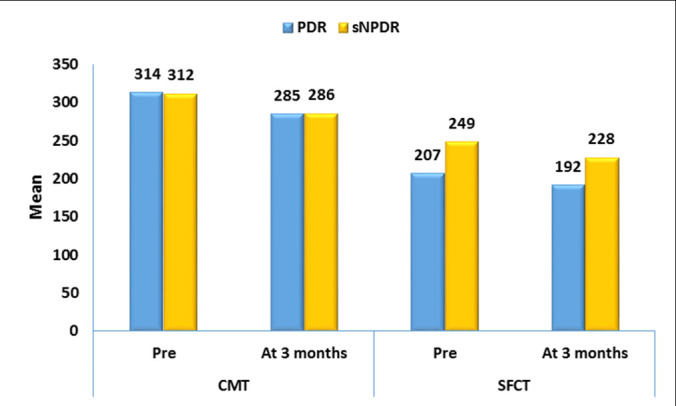
Bar chart showing a significant thinning in the mean CMT and SFCT in sNPDR (27 eyes) subgroup versus the baseline after PRP (p <0.001). In the subgroup of PDR who underwent PRP (17 eyes), the mean CMT and SFCT were reduced from baseline of 314 ± 87 µm and 207 ± 48 µm to 285 ± 88 µm and 192 ± 49 µm 3 months after PRP, respectively (p<0.001)

**Fig. 4:**
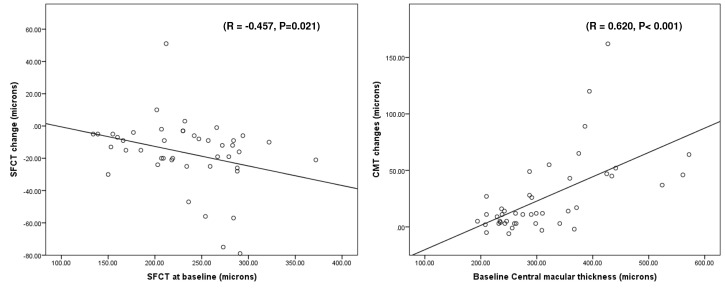
Scatter plots showing that the SFCT changes correlated significantly with the baseline SFCT (left plot), and the changes in the CMT correlated significantly with baseline CMT as well (right plot)

No significant association between changes in CMT and SFCT (P = 0.641) were observed. However, stepwise multiple regression analysis disclosed that the mean SFCT at baseline (R = -0.457, P=0.021, **Fig. 4**) was significantly associated with the mean changes of SFCT. Likewise, pre-PRP CMT was the only parameter that correlated with CMT changes (R = 0.620, P < 0.001, **Fig. 4**). The severity of DR showed significant negative association with baseline SFCT (R = −0.380, P = 0.011).

## Discussion

Management of different ocular disorders has been revolutionized due to the tremendous advances in OCT technology [**24**,**25**,**29**]. This cohort of 44 eyes with a mean follow-up duration of 3 ± 0.6 months after the last session of the PRP represented the short-term longitudinal changes in the CMT and SFCT using SS-OCT. In agreement with some of the previously published reports [**7**,**8**,**10**-**12**], the average SFCT and CMT seemed to decrease in sNPDR and PDR after PRP treatment.

Several hypotheses were anticipated to elucidate SFCT reduction after PRP. PRP-induced RPE damage with subsequent diminution in the VEGF production and decreased dilation of choroidal vessels has been proposed [**7**]. Another theory is that the PRP-induced reduction of the choroidal perfusion could be attributed to choroidal autoregulation to enhance the hypoxic inner retina [**7**]. Gradual choroidal thinning has been observed over 1 year follow-up period, suggesting the long-term effect of PRP on choroidal flow [**8**]. Kang hypothesized that PRP-induced reduced choroidal pachymetry may prevent further progession of DR [**8**]. 

Although SFCT may increase initially after PRP as a result of PRP-induced choriocapillaris damage with subsequent choroidal vasodilation [**7**,**26**], a longer follow-up showed reduction of SFCT at 2-3 months [**7**].

On the contrary, Zhu [**9**] and Roohipoor [**13**] reported a thickening in the SFCT of up to 12 and 24 weeks after PRP, respectively. This observation was explained by PRP-induced inflammation with subsequent cytokines surge and increased choroidal perfusion [**27**,**28**]. Discrepancies between different reports may be attriburted to different patients’ population and inclusion criteria. Some studies recruited patients with co-existing DME and others included patients who previously underwent PRP. 

Our study demonstrated a significant decline in the average CMT at POM#3. However, there were no significant variations among the groups in SFCT and CMT at POM#3. Our study revealed a high accuracy of the CMT and SFCT measurements obtained with Topcon Advanced Boundary Software. An excellent repeatability was found for repeated measures of CMT and SFCT. Subgroup analysis of both sNPDR and PDR patients showed that the mean SFCT of patients with sNPDR was thicker than that of PDR before and at POM#3; while, non-significant variations in the average CMT between the 2 groups at both visits were reported.

Limitations of the study include the small cohort size and short follow-up duration after PRP. Further studies with a larger cohort and longer follow-up duration are required to replicate our conclusions. However, our study is notable for being prospective in nature contrasted to several published reports. We also assessed the variations in CMT, which have not been comprehensively studied before.

To conclude, our study supports the published evidence, demonstrating a significant decline of SFCT and CMT 3 months after PRP treatment in either sNPDR or PDR. Besides, SS-OCT is a reliable tool for quantification of SFCT in diabetic retinopathy with excellent accuracy.
